# End-to-end neural system identification with neural information flow

**DOI:** 10.1371/journal.pcbi.1008558

**Published:** 2021-02-04

**Authors:** K. Seeliger, L. Ambrogioni, Y. Güçlütürk, L. M. van den Bulk, U. Güçlü, M. A. J. van Gerven

**Affiliations:** 1 Radboud University, Donders Institute for Brain, Cognition and Behaviour, Nijmegen, The Netherlands; 2 Max Planck Institute for Human Cognitive and Brain Sciences, Leipzig, Germany; Harvard University, UNITED STATES

## Abstract

Neural information flow (NIF) provides a novel approach for system identification in neuroscience. It models the neural computations in multiple brain regions and can be trained end-to-end via stochastic gradient descent from noninvasive data. NIF models represent neural information processing via a network of coupled tensors, each encoding the representation of the sensory input contained in a brain region. The elements of these tensors can be interpreted as cortical columns whose activity encodes the presence of a specific feature in a spatiotemporal location. Each tensor is coupled to the measured data specific to a brain region via low-rank observation models that can be decomposed into the spatial, temporal and feature receptive fields of a localized neuronal population. Both these observation models and the convolutional weights defining the information processing within regions are learned end-to-end by predicting the neural signal during sensory stimulation. We trained a NIF model on the activity of early visual areas using a large-scale fMRI dataset recorded in a single participant. We show that we can recover plausible visual representations and population receptive fields that are consistent with empirical findings.

## Author summary

We propose a method for data-driven estimation of computational models, representing neural information processing between different cortical areas. We demonstrate this method on the largest single-participant naturalistic fMRI dataset recorded to date. By training a simplified model of the visual system we show that biologically plausible computations emerge in the training process, yielding a new approach to understanding information processing in neural systems. The approach is applicable to other sensory or imaging modalities, thus providing a general way to computational modeling in cognitive neuroscience.

This is a *PLOS Computational Biology* Methods paper.

## Introduction

Uncovering the nature of neural computations is a major goal in neuroscience [1]. It may be argued that true understanding of the brain requires the development of *in silico* models that explain the activity of biological neurons in terms of information processing. We refer to this idea as *neural system identification* [2, 3]. In cognitive terms, information processing can be understood as using internal representations of environments with the goal of generating behaviour.

The predominant approach for uncovering these representations is to use predefined nonlinear features derived from the stimulus as a hypothesis for predicting measured neural responses [4–6]. Using this approach, in visual and auditory domains the best results so far have been obtained by using convolutional (or deep) neural networks (DNNs) [6–15]. DNNs process input through a sequence of layers with linear and nonlinear transformations, and learn local features and maps of these features through the convolution operation. Each layer of a DNN encodes increasingly more complex abstractions of the original input. However, using this approach DNNs have to be trained for solving manually defined tasks such as object classification on specific data bases. Consequently, the resulting DNN feature representations are biased towards their specific objective function.

An alternative approach is to directly estimate hierarchical representations from neural data. This idea has been used to reveal mechanisms of neural information processing in biological systems [13, 16–24]. However, most of these ideas have been applied within individual brain regions (most frequently within V1) and using invasive data. In the area of human visual perception across multiple areas, the most related approach is *Representational Distance Learning* [25, 26], which uses representational dissimilarity matrices estimated within visual areas as an element of the training objective of a convolutional neural network modeling these areas. Recent approaches use the prediction of neural measurements directly for learning to separate the location and features that voxels respond to [13, 17, 18, 24, 27, 28]. This manuscript expands on this work, proposing a novel approach for neural system identification, referred to as *neural information flow* (NIF). NIF generalizes existing approaches, allowing estimating neural information processing systems from individual cortical areas up to the whole-brain level.

Similar to DNN encoding models, the information processing hierarchy is expressed as a multi-layer neural network. However, the layers of NIF models have a one-to-one correspondence to biological neural populations (such as V1), and all neural network parameters are solely trained with the objective function of predicting brain activity measured in response to input stimuli. Using this method, training is expected to learn spatiotemporal neural representations of the sensory input inside the corresponding population, and learn to derive the underlying flow of information processing. In neurobiological terms, DNN nodes can be interpreted as the activation of a cortical column responsive to a specific local feature, such as a Gabor wavelet in V1. The cascade of convolutional layers can be interpreted as the topologically organized connectivity between brain regions.

Convolutional layer activity is linked to neural measurement units through unit-wise observation models that are trained jointly with the other network parameters. The choice of measurement unit (e.g. cellular, voxels, behavioural) in the NIF framework is arbitrary, and measurements can be combined. In case of functional magnetic resonance imaging (fMRI) from a visual experiment, each voxel learns its spatial receptive field and local peak of the hemodynamic response; and the preferred convolutional features (channels) of its underlying information processing units.

In this manuscript we outline the principles and methodology of NIF with a simplified model of the visual system. Using a large fMRI dataset acquired under stimulation with naturalistic video we demonstrate that the model is capable of generating realistic brain measurements, and that the computations learned inside the model are biologically meaningful. We expect that these ideas will guide the development of a new family of computational models that allow uncovering the principles of neural computations in biological systems.

## Methods

### Ethics statement

Data collection was approved by the local ethical review board (CMO regio Arnhem-Nijmegen, The Netherlands, CMO code 2014-288 with amendment NL45659.091.14) and was carried out in accordance with the approved guidelines. For each session written formal consent was obtained from the participant. All specifics of the data set are described in a separate manuscript accompanying the data publication [29].

### Neural information flow

**Fig 1:**
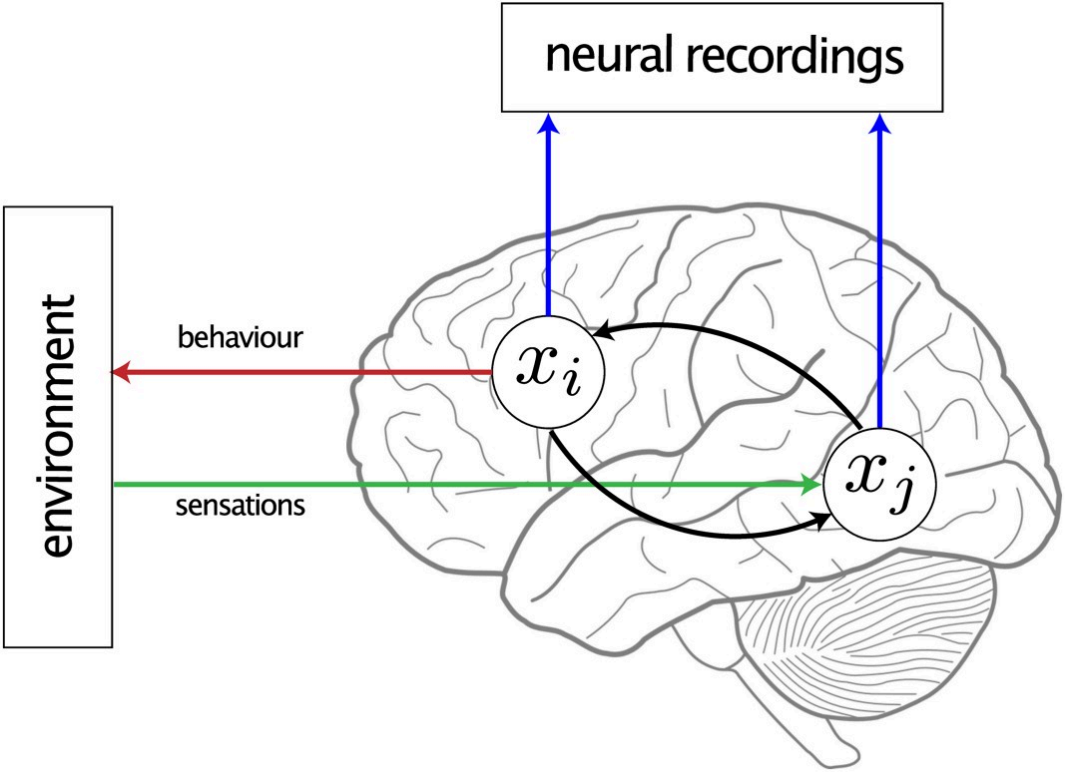
The philosophy underlying neural information flow. NIF models define synthetic brains that model information processing in real brains. They are specified in terms of mutually interacting neuronal populations (white discs) that receive sensory input (green) and give rise to measurements of neural activity (blue) and/or behavior (red). In practice, NIF models may consist of up to hundreds such interacting populations. They can be estimated by fitting them to neurobehavioral data acquired under these tasks. By analyzing NIF models, we can gain a mechanistic understanding of neural information processing in real brains and how neural information processing relates to phenomenology.

The purpose of a NIF model is to capture the neural computations that take place within and between neuronal populations in response to sensory input. The general philosophy of NIF is outlined in Fig 1. The core of a NIF model is a deep modular neural network architecture where individual neuronal populations are modeled using neural network modules that transform afferent input into efferent output. The connectivity between populations is captured by convolutional layers which model the topographically organized information exchange between neuronal populations. Finally, population activity is used to predict observed measurements through factorized observation models. Model parameters are estimated by fitting the neural signals measured during sensory stimulation. Specifically, the NIF model receives the same sensory input that is presented to the participant and predicts the measurements of all brain regions of interest. Model components are trained end-to-end using stochastic gradient descent to minimize the error in voxel-specific measurement predictions. In the following we describe the NIF components in more detail.

#### Modeling sensory input and neural representations

Sensory input is modeled using a four-dimensional tensor $N\in {\mathbb{R}}^{N_{c}\times N_{t}\times N_{x}\times N_{y}}$ whose array dimensions represent input channels *c*, time *t* and spatial coordinates (*x*, *y*) respectively. For example, the input channels can be the RGB components of a visual stimulus or the photoreceptor responses of a retinal model. In our experiments, we model grayscale images using a single luminance channel (*N*_*c*_ = 1). We used temporal windows of 2.1s, resulting in 48 frames (*N*_*t*_ = 48). Analogously, the representations of the sensory input encoded in each brain region are modeled using four-dimensional tensors. The feature maps **N**[*c*, :, :, :] of these neural tensors encode neural processing of specific sensory stimulus features such as oriented edges or coherent motion. Consequently, a tensor element can be interpreted as the response of one cortical column. Under the same interpretation, cortical hyper-columns are represented by a sub-tensor **N**[:, :, *x*, *y*] storing the activations of all the columns that respond to the same spatial location.

#### Modeling directed connectivity and information flow

We model the directed connectivity between brain regions using spatiotemporal convolutions. The spatial weights model the topographically organized synaptic connections while the temporal component models synaptic delays. Using this setup, we can model how neural populations respond to sensory input as well as to each other. Note that to enforce causality of the neural responses, the temporal filters should be causal, meaning that the only non-zero weights correspond to past time points. However, this assumption can be dropped when the time scale of our observations is much slower than that of the underlying temporal dynamics (as in BOLD data).

Let $\tilde{N}$ denote the concatenation of afferent inputs **N**_1_, …, **N**_*N*_ along the feature dimension and let ⋆ denote the convolution operation. We define the activation of the *j*-th brain area as a function of its afferent input as follows:

$$\begin{array}{c} N_{j}=f_{j}\left(N_{1},\ldots ,N_{N}\right)=f(\tilde{N}⋆W_{j}+B_{j})\;, \end{array}$$ (1)

where **f** is the element-wise application of a sigmoid activation function followed by downsampling using an average pooling operation, **W**_*j*_ is a synaptic weight kernel and **B**_*j*_ is a bias term. Initial testing indicated more stable convergence using sigmoid activation functions compared to ReLU activation functions.

### Modeling observable signals

NIF models are estimated by linking neural tensors to observation models that capture indirect measurements of brain activity. Observations are represented using tensors **Y** that store measurable responses. The observation model expresses the predicted measurements as a function of the activity of the latent tensors:

$$\begin{array}{c} Y=g(N_{1},\ldots ,N_{N})+\epsilon \;, \end{array}$$ (2)

where ***ϵ*** is measurement noise. The exact form of **g** depends on the kinds of measurements that are being made. Neuroimaging methods such as fMRI, single- and multi-unit recordings, local field potentials, calcium imaging, EEG, MEG but also motor responses and eye movements are observable responses to afferent input and can thus be used as a training signal. Note that the same brain regions can be observed using multiple observation models, conditioning them on multiple heterogeneous datasets at the same time. This provides a solution for multimodal data fusion in neuroscience [30]. In this paper, we focus on modeling blood-oxygenation-level dependent (BOLD) responses obtained for individual voxels using fMRI. In this case, we can consider the voxel responses separately for each region, such that we have **Y**_*i*_ = **g**_*i*_(**N**_*i*_) + ***ϵ*** for each region *i*. Let $Y_{i}\in {\mathbb{R}}^{K\times T}$ denote BOLD responses of *K* voxels acquired over *T* time points for the *i*-th region. Our observation model for the *k*th voxel in that region is defined as

$$\begin{array}{c} Y_{i}\left[k,t+\Delta_{t}\right]=b_{k}+\sum_{c,\tau ,x,y}N_{i}\left[c,\tau ,x,y\right]U_{i}\left[c,\tau ,x,y,k\right]+\epsilon \left[k\right]\;, \end{array}$$ (3)

where **N**_*i*_ contains neural network activations to the stimulus frames presented in preceding video chunks, relative to time *t*, *b*_*k*_ is a voxel-specific bias, *ϵ*[*k*] is normally distributed measurement noise and Δ_*t*_ is a temporal shift of the BOLD response that is used to take into account a default offset in the hemodynamic delay (4.9 s in our experiments). Every brain region can be observed using a function of the form shown in Eq (3).

#### Factorized observation models

To simplify parameter estimation and facilitate model interpretability we use a factorized representation of **U** (also see [17]. That is,

$$\begin{array}{c} U\left[c,t,x,y,k\right]=U_{c}\left[c,k\right]U_{t}\left[t,k\right]U_{s}\left[x,y,k\right]\;, \end{array}$$ (4)

where *k* is denotes the voxel index. Here, **U**_*c*_[⋅, *k*] are the feature loadings that capture the sensitivity of a voxel to specific input features, **U**_*t*_[⋅, *k*] is the temporal profile of the observed BOLD response of a voxel and **U**_*s*_[⋅, ⋅, *k*] is the spatial receptive field of a voxel. Hence, the estimated voxel-specific observation models have a direct biophysical interpretation.

We further facilitate parameter estimation by using a spatial weighted low-rank decomposition of the spatial receptive field:

$$\begin{array}{c} U_{s}\left[x,y,k\right]\approx \sum_{r=1}^{R}a_{k,r}U_{x,r}\left[x,k\right]U_{y,r}\left[y,k\right]\;. \end{array}$$ (5)

Here, *a*_*k*,*r*_ are rank amplitudes that are constrained to be positive using a softplus transformation. We used *R* = 4 in our experiments. The rank limits the complexity of the spatial observation model. Rank one models can estimate unimodal receptive fields. However, a small number of voxels have nonclassical receptive fields that respond to multiple parts of the input space, for which more degrees of freedom are needed. To further stabilize the model and obtain localized and positive spatiotemporal receptive fields, we apply a softmax nonlinearity to the columns of **U**_*t*_, **U**_*x*_ and **U**_*y*_. That is, the elements *u*_*i*_ of each column vector **u** of these matrices are given by

$$\begin{array}{c} u_{i}=\sigma_{i}\left(v\right)=\text{exp}\left(v_{i}\right)/\sum_{j}\text{exp}\left(v_{j}\right)\;, \end{array}$$ (6)

where the *v*_*i*_ are learnable parameters.

#### Model estimation

Once the architecture of the NIF model is defined, synaptic weights and observation model parameters can be estimated by minimizing a loss using gradient descent via backpropagation. Let ${\text{Y}}_{i}^{t}$ and ${\hat{\text{Y}}}_{i}^{t}={\text{g}}_{i}\left({\text{N}}_{i}\right)$ denote the observed and predicted measurements for the *i*th region relative to the *t* measurement (BOLD volume). The loss is given by the squared error per region summed over regions and across measurements:

$$\begin{array}{c} L=\sum_{t,i}{\left({\hat{Y}}_{i}^{t}-Y_{i}^{t}\right)}^{2}\;. \end{array}$$ (7)

Note that, since the model couples neuronal populations, region-specific estimates are constrained by one another and consequently make use of all observed data. Our approach was implemented in the Chainer framework for automatic differentiation [31].

### Experimental validation

To demonstrate the capabilities of the NIF framework, we estimated and tested a simple visual system model using a unique large-scale functional MRI dataset collected while one participant was exposed to almost 23 hours of complex naturalistic spatiotemporal stimuli. Specifically, we presented episodes from the BBC series *Doctor Who* [32].

#### Stimulus material

A single human participant (male, age 27.5) watched 30 episodes from seasons 2 to 4 of the 2005 relaunch of *Doctor Who*. This comprised the training set which was used for model estimation. Episodes were split into 12 min chunks (with each last one having varying length) and presented with a short break after every two runs. The participant additionally watched repeated presentations of the short movies *Pond Life* (five movies of 1 min, 26 repetitions) and *Space / Time* (two movies of 3 min, 22 repetitions), in random permutations and after most episodes. They were taken from the series’ next iteration to avoid overlap with the training data. This comprised the test set which was used for model validation.

#### Data acquisition

We collected 3T whole-brain fMRI data. It was made sure that the training stimulus material was novel to the participant. Data were collected inside a Siemens 3T MAGNETOM Prisma system using a 32-channel head coil (Siemens, Erlangen, Germany). A T2*-weighted echo planar imaging pulse sequence was used for rapid data acquisition of whole-brain volumes (64 transversal slices with a voxel size of 2.4 × 2.4 × 2.4 mm^3^ collected using a TR of 700 ms). We used a multiband-multi-echo protocol with multiband acceleration factor of 8, TE of 39 ms and a flip angle of 75 degrees. The video episodes were presented on a rear-projection screen with the Presentation software package, cropped to 696 × 732 pixels squares so that they covered approximately 20 degrees of the vertical and horizontal visual field. The participant’s head position was stabilized within and across sessions by using a custom-made MRI-compatible headcast, along with further measures such as extensive scanner training. The participant had to fixate on a fixation cross in the center of the video. At the beginning of every break and after every test set video a black screen was shown for 14 s to record the fadeout of the BOLD signal after video presentation stopped. The black screen stimuli of these periods were omitted in the present analysis. In total this leaves us with approximately 118.000 whole-brain volumes of single-presentation data, forming our training set (used for model estimation) and 1.032 volumes of resampled data, forming our test set (used for model evaluation). We decided to use the whole test set, including the second half with the slight vertical elongation.

#### Data preprocessing

Minimal BOLD data preprocessing was performed using FSL v5.0. Volumes were first aligned within each 12 min run to their center volume (run-specific reference volume). Next, all run-specific reference volumes were aligned to the center volume of the first run (global reference volume). The run-specific transformations were applied to all volumes to align them with the global reference volume. The signal of every voxel used in the model was linearly detrended, then standardized (demeaning, unit variance) per run. Test set BOLD data was averaged over repetitions to increase signal to noise ratio, and as a final step the result was standardized again. A fixed delay of 7 TRs (4.9 s) was used to associate stimulus video segments with responses and allow the model to learn voxel-specific HRF delays within **U**_*t*_. With the video segments covering 3 TRs starting from the fixed delay, the BOLD signal corresponding to a stimulus is thus expected to occur within a time window of 4.9 s to 7.0 s after the onset of the segment. As there were small differences between frame rates in the train and test sets we transcoded the stimulus videos to a uniform frame rate of 22.86 Hz (16 frames per TR) for training the example model. To reduce model complexity we downsampled the videos to 112 × 112. As the model operates on three consecutive TRs, the training input size was 112 × 112 × 48. The stimuli were converted to grayscale [33] prior to presenting them to the model. Otherwise stimuli were left just as they were presented in the experiment.

#### Model architecture

**Fig 2:**
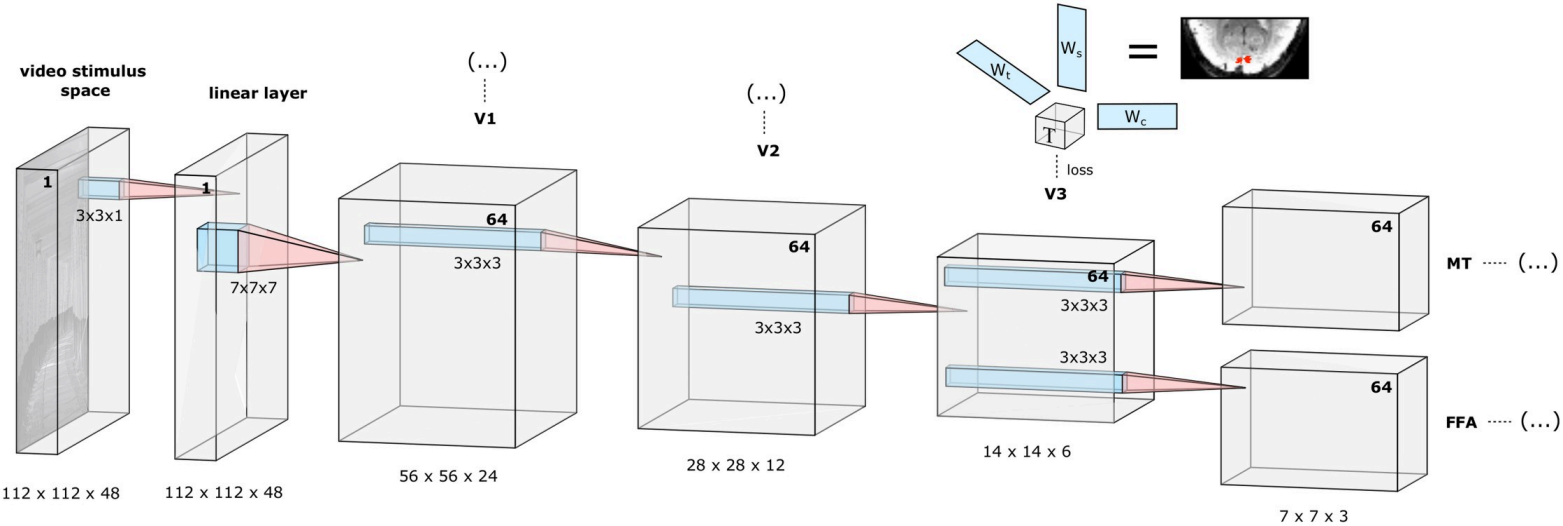
The described NIF architecture, a simplified feed-forward model of early visual areas. Underneath the tensors resulting from the 3D convolution operations we state the size of each input space (*x* × *y* × *t*) to the next layer. The number of feature maps in each input space is printed in boldface, with the stimulus (input) space consisting of a single channel. The input to the network are 3D stimulus video segments consisting of 3 × 16 frames (covering three TRs of 700 ms each), aligned with the hemodynamic response by applying a fixed delay of 7 TRs. The first convolutional layer is not attached to a region observation model, but is a single-channel linear spatial convolution layer. It serves as a learnable linear preprocessing step that accounts for retinal and LGN transformations. Convolutional kernel sizes are 7 × 7 × 7 in the second convolutional layer (leading to the V1 tensor), and 3 × 3 × 3 for all other layers. After every convolution operation (except for the linear layer) we apply a sigmoid nonlinearity and spatio-temporal average pooling with 2 × 2 × 2 kernels. Before entering the **U**_*t*_ observation models the temporal dimension is average pooled so that each point *t* covers one TR. All weights in this model (colored blue) are learned by backpropagating the mean squared error losses from predicting the BOLD activity of the observed voxels. The voxel-specific observation models consisting of the spatiotemporal weight vectors **U**_*s*_ and **U**_*t*_ and the feature observation model **U**_*c*_ enable the end-to-end training of the model from observational data.

We implemented a purely feed-forward architecture for modeling parts of the visual system (V1, V2, V3, FFA and MT). The used architecture is illustrated in detail in Fig 2. FFA and MT have their own tensors originating from V3 to allow for a simplified model of the interactions between upstream and downstream areas. We intentionally used a simplified model to focus on demonstrating the capabilities of the NIF framework. To model LGN output, we used a linear layer consisting of a single 3 × 3 × 1 spatial convolutional kernel. The NIF model was trained for 11 epochs with a batch size of 3, using the Adam optimizer [34] with learning rate *α* = 5 × 10^−4^. Weights were initialized with Gaussian distributions scaled by the number of feature maps in every layer [35].

## Results

In this paper we focus on the processing of visual information. In the following, we show that a NIF model uncovers meaningful characteristics of the visual system.

### Accuracy of response predictions

**Fig 3:**
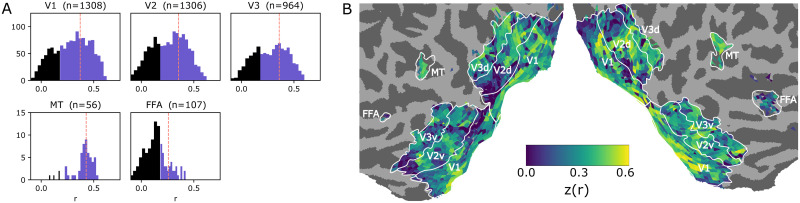
Voxel-wise correlations. A. Histograms of voxel-wise correlations between predicted and observed BOLD responses on the test set in different observed brain regions. The vertical line marks the median. The blue area shows the significantly predicted voxels. B. Cortical flatmap of the distribution of all correlations across the visual system. For the map we applied a Fisher z-transform to facilitate linear visual comparison of correlation magnitudes.

After training the NIF model, we tested its accuracy on the test set. We observed that BOLD responses in a majority of voxels in each brain region could be predicted by the model (tested for significance with *p* < 0.01, Bonferroni-corrected over the total number of gray matter voxels). This is illustrated in Fig 3, showing voxel-wise correlations between predicted and observed test data per region. The results show that the NIF model generates realistic brain activity in response to unseen input stimuli. The larger correlations in area MT could be explained by its motion-sensitivity, which can be strongly driven by the employed video stimulus and can be modeled well using a relatively straightforward motion energy model [36].

### Visualization of learned representations

In this subsection we examine the features of the external stimulus that are encoded in our trained model of the visual system. We will begin with an analysis of the first layers, LGN and V1, whose features can be visualized by plotting the weights of the convolutional kernels. We will then show visualization of higher order regions using a more sophisticated preferred input analysis.

#### Linear feature analysis

**Fig 4:**
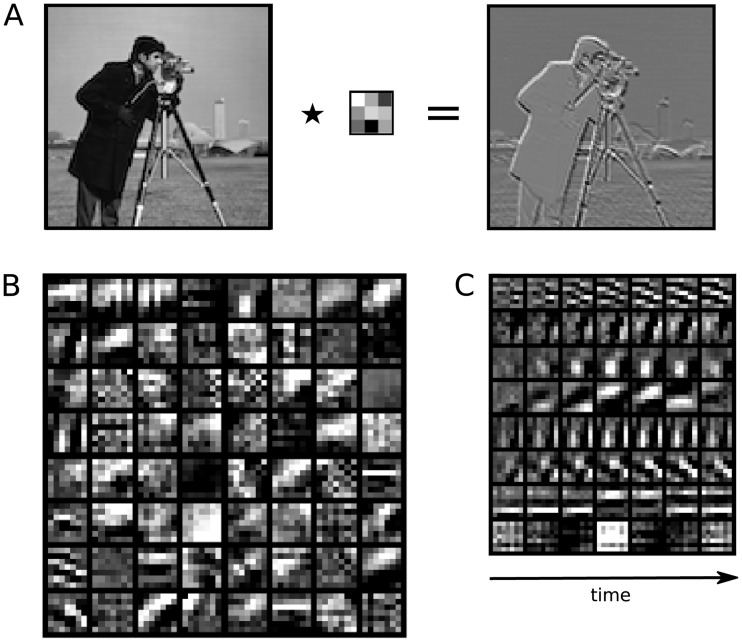
Stimulus features derived by the NIF model. A. Learned linear preprocessing showing that the estimated kernel extracts edges from the original input image. B. The 64 spatial features estimated from neural data for area V1 (frame three out of seven). C. Visualization of seven of these features across the temporal dimension. For visualization, feature weights were clipped at the extremes and all weights were globally rescaled between zero and one. See S1 Video for the animated version.

For the first layers of the model, before the application of nonlinear transformations, neural network features can be inspected by visualizing the learned weights. A linear single-channel spatial layer was used to represent the transformation of the visual input at the retinal/LGN stage, before it enters the visual cortex [37, 38]. Fig 4A shows the estimated kernel as well as the resulting image transformation when applying this kernel to the input. As we can see, the linear kernel learns to extract edges at different orientations, as well as (albeit weaker) luminance. The result is strikingly similar to that of analytical ZCA whitening, however emphasizes edges further. When learning two linear kernels instead of one (as in our model), one kernel learns to extract luminance while the other extracts edges. This is likely to be a reflection of the independence of luminance and contrast information in natural images and in LGN responses [39]. We can also visualize the feature detectors that determine the responses of V1. Fig 4B shows the 64 channels learned by the neural tensor connected to V1 voxels. Several well-known feature detection mechanisms of V1 arise, such as Gabor-like response profiles [40]. As shown in Fig 4C, several of these feature detectors also show distinct dynamic temporal profiles, reflecting the processing of visual motion [16].

#### Preferred input analysis

Feature sensitivities in DNNs can only be investigated by directly plotting the learned weights before non-linearities are applied. For higher order regions, neural network interpretability methods need to be used. For instance, we can gain insight into the nature of the representations of higher order regions by visualizing which stimulus properties best drive simulated neural responses in a particular brain region. To this end, we estimated the gradient that leads to an increase in activity in individual target voxels, and used this gradient to modify the input such as to optimally drive the voxel response, starting from a three-dimensional white noise input. The technique is similar to [41], and similar in spirit to [42–44]. The basic approach was originally proposed in [45].

Let *I*_*t*,*x*,*y*_ denote the pixel intensity for the *t*th frame at spatial location (*x*, *y*). The size of *I* matches the input dimension of 48 × 112 × 112 and is initialized with random values in the same range as the original input.

The analysis was performed only for those voxels for which the correlation between predicted and observed responses exceeded 0.4 on the test set. Let **y** = (*y*_1_, …, *y*_*K*_) such that **y** denotes the activity of all voxels in a specific ROI and *y*_*k*_ denote the response of the *k*th target voxel (the voxel that’s activity should be maximized). The objective is to optimize

$$\begin{array}{c} \sigma_{k}\left(y\right)=\frac{\text{exp}(y_{k})}{\sum_{i}\text{exp}\left(y_{i}\right)}\;. \end{array}$$ (8)

and

$$\begin{array}{c} \gamma_{k}\left(y\right)=y_{k}\;. \end{array}$$ (9)

That is, we modify the input such as to maximize the activity of the *k*th voxel *y*_*k*_, while suppressing the responses of all other voxels in the same ROI *y*_*v*_ using a softmax nonlinearity. This leads to an high amplitude both in absolute value and relative to the other voxels within a ROI.

We further regularize the input using an *ℓ*_1_ loss on all components (pixel values) of *I*. The *ℓ*_1_ leads to the suppression of noise in the image, which otherwise easily occurs in this optimization process.

The objective is thus to minimize

$$\begin{array}{c} -\text{log}\left(\sigma_{k}\left(y\right)\right)-\gamma_{k}\left(y\right)+\lambda \ell_{1}\;, \end{array}$$ (10)

with λ = 10^−7^ for FFA and MT and λ = 10^−6^ in other ROIs.

A standard SGD optimizer was used together with an adaptive learning rate (starting value *η* = 10^7^, reduction factor 0.8 after 5 iterations with no change) to optimize the stimuli. The iteration was stopped when no pixel changed more than 10^−3^ within 50 optimization steps.

As our video stimuli were square one way this optimization structure could exploit the objective was to cover the whole image with 45° oriented moving bars, as diagonals across the image would be the optimal way to create most energy within the input. We could work around this issue by retraining the NIF model with a circular aperture superimposed on the input videos. During preferred input optimization the aperture region was excluded by setting its gradients to 0. A similar effect could occur at small frequencies due to standard convolutional filters in current neural networks operating within square receptive fields. This can only be solved by adopting non-squared convolutional filters.

**Fig 5:**
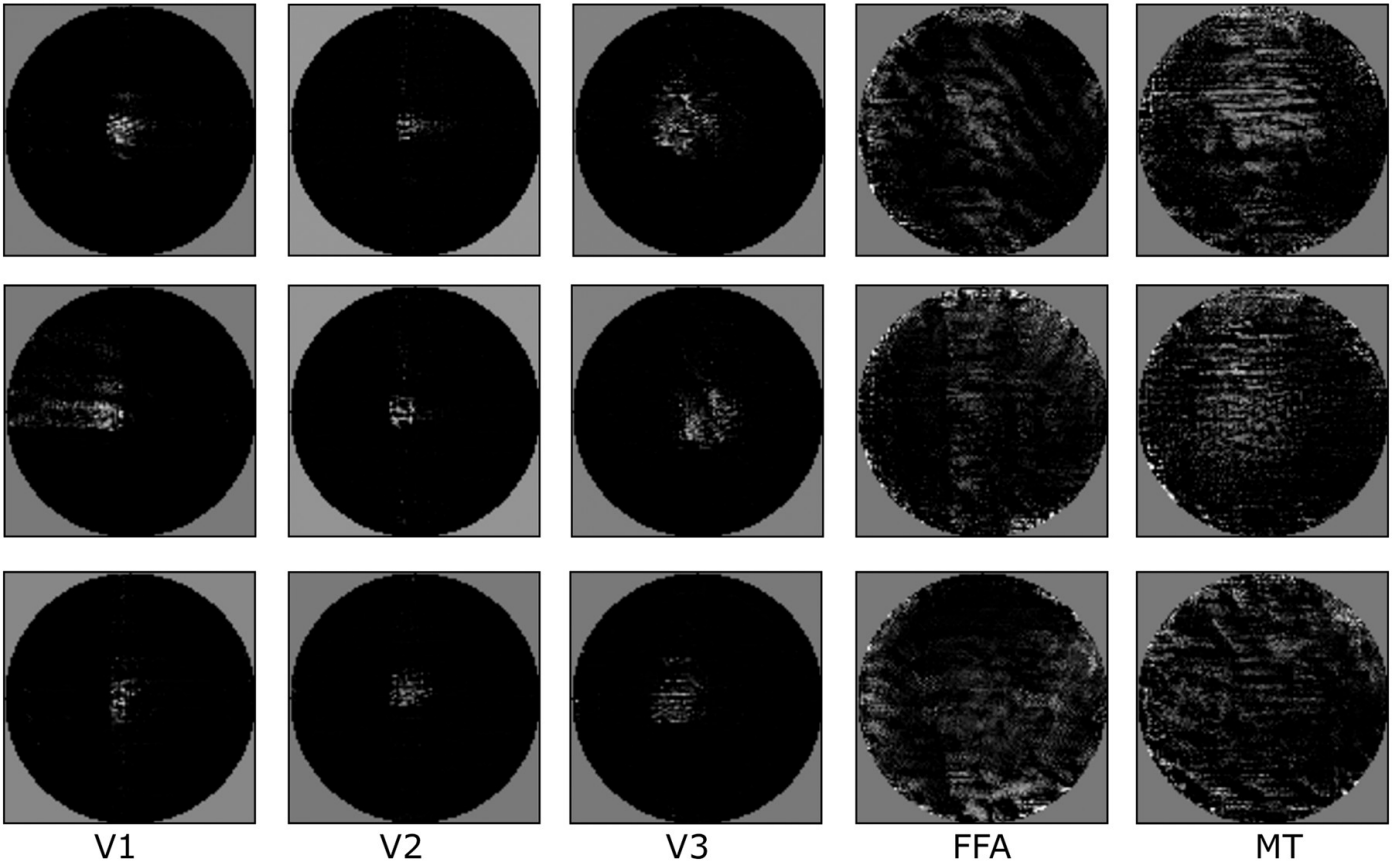
Examples of preferred inputs that maximize simulated voxel responses in different brain regions. Static frames from preferred inputs for three different voxels in the modeled ROIs. See S2 Video for observing the behaviour of these preferred inputs over time.

The results for different areas can be seen in Fig 5. All preferred inputs show superimposed moving wavelets at different orientations and frequencies. For V1, V2 and V3 they are constrained to their receptive fields. MT shows large circular fields of superimposed frequencies. FFA also shows larger regions of superimposed frequencies with circular dropouts.

The preferred inputs of V1, V2 and V3 are plausible, while the derivations for the higher order regions are difficult to interpret. Note that our example architecture is not biologically plausible, so this analysis should be read as a demonstration of the option of deriving preferred inputs of voxels rather than as a new insight into our cognition.

As stated at the beginning of this section, a different approach for visualizing what has been learned from the ROI data would be deriving what the higher order convolutional neural network channels represent, rather than observing what individual voxels prefer, i.e. a visualization of channels akin to Fig 4, but for higher order regions. This would avoid the superimposing nature of the voxelwise preferred images. This is a topic of research currently investigated by convolutional neural network interpretability, and not satisfactorily solved yet [46, 47].

### Receptive field mapping

**Fig 6:**
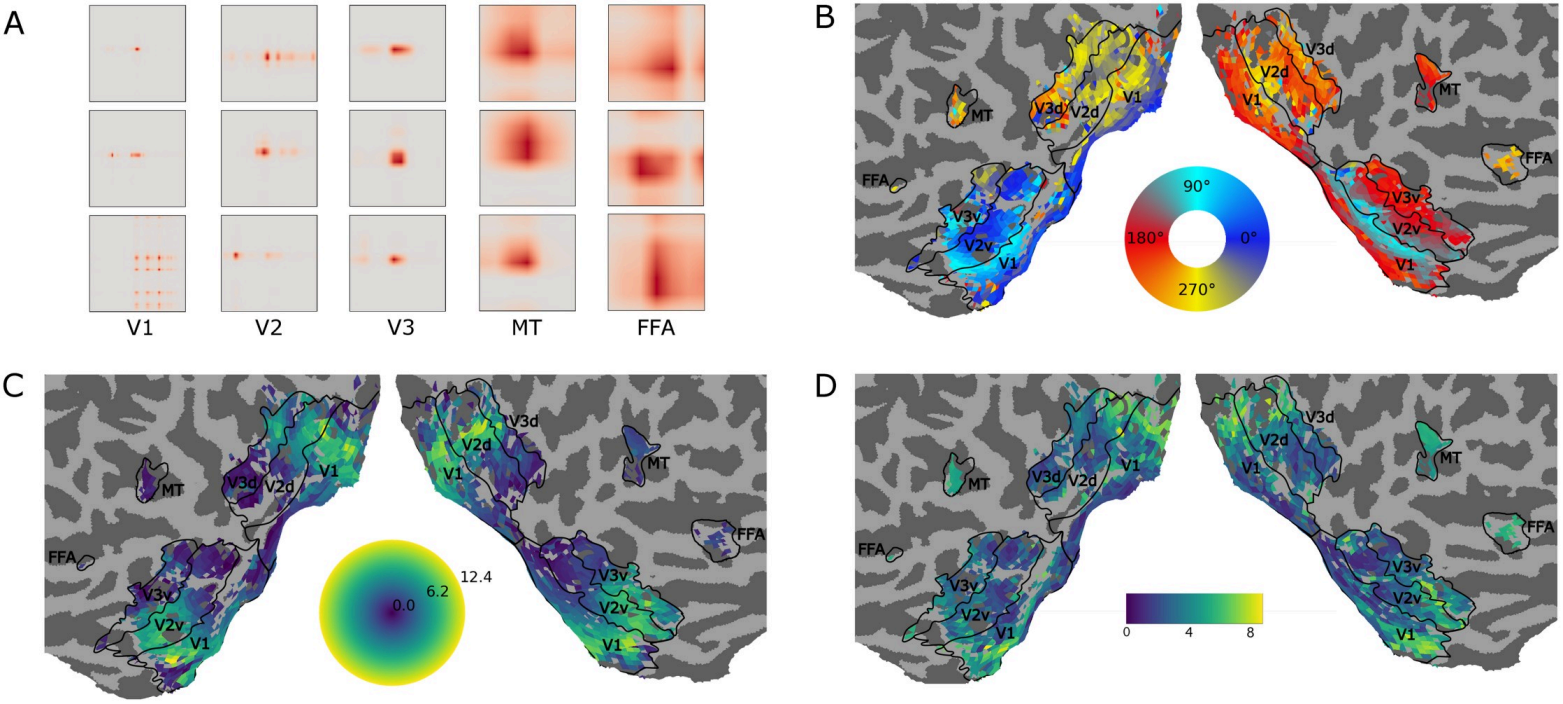
Receptive field maps. A. Various spatial receptive fields in video pixel space **U**_*s*_ learned for different ROIs within our framework. Most estimated spatial receptive fields are unipolar. B-D. Basic retinotopy that arose in the voxel-specific spatial observation matrix **U**_*s*_ within the NIF model. B. Polar angle. C. Eccentricity. D. Receptive field size.

We examined whether the retinotopic organization of the visual cortex can be recovered from the spatial observation models [48]. Here, **U**_*s*_ represents spatial receptive field estimates for every voxel. Some of these voxel-specific receptive fields are shown in Fig 6A. The model has primarily learned classical local unimodal population receptive fields, but also more complex non-classical response profiles. This matches the expectation that population responses as inferred from neuroimaging data are not necessarily restricted to unipolar receptive fields. The model can be further constrained in case unipolar responses are expected (see [43] for a possible approach).

To check that the NIF model has indeed captured sensible retinotopic properties, we determined the center of mass of the spatial receptive fields and transformed these centers to polar coordinates using the central fixation point as origin. Sizes of the receptive fields were estimated as the standard deviation across **U**_*s*_, using the centers of mass as mean. Due to the pooling operations and convolutional processing, the **U**_*s*_ for each voxel had to be rescaled to the original input size to perform this operation. Voxels whose responses could not be significantly predicted were excluded from this analysis. Fig 6 shows polar angle (B), eccentricity (C) and receptive field size (D) for early visual system areas observed by our model. Maps were generated with pycortex [49]. Note that the boundaries between visual areas V1, V2 and V3 have been estimated with data from a classical wedge and ring retinotopy session. As can be seen, reversal boundaries align well with the traditionally estimated ROI boundaries. The larger eccentricity and increase in receptive field size (C) matches the expected fovea-periphery organization as well. Our results thus indicate that the NIF framework allows the estimation of accurate retinotopic maps from naturalistic videos.

### Further properties of observational models

**Fig 7:**
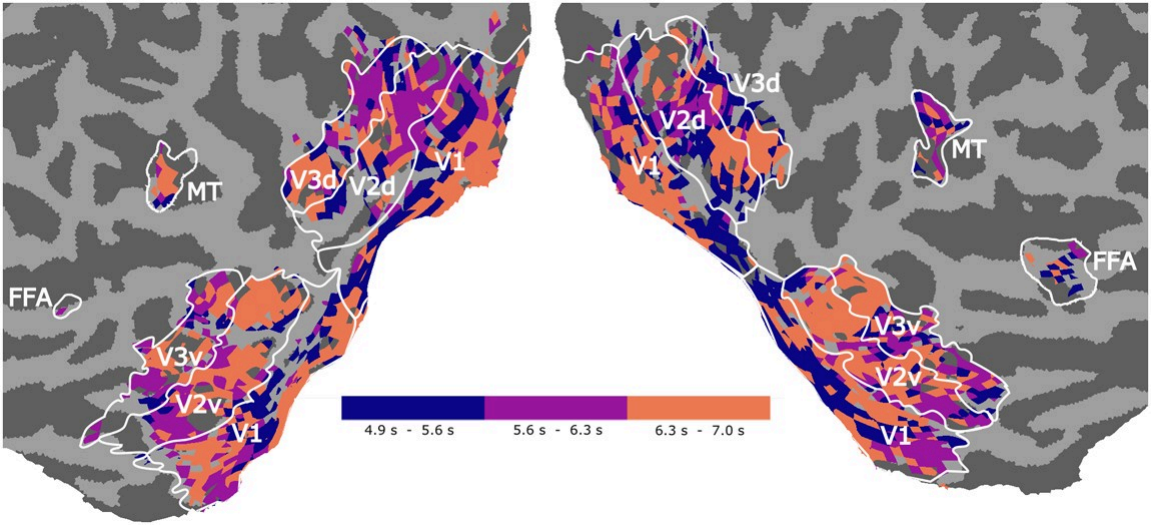
Differences in hemodynamic delay extracted from U_*t*_. For every voxel *k* we see the delay encoded in **U**_*t*_[*t*, *k*] that has the maximal weight.

Recall that our model aims to predict the observed BOLD response from a spatiotemporal stimulus. We can obtain a rough estimate of the peak of the BOLD response by determining for each voxel the delay *t* that has the maximal weight **U**_*t*_[*t*, *k*] assigned. Fig 7 shows the distribution of these delays across cortex, providing an insight into spatial differences in the hemodynamic response function. Results show a consistent slowing of the HRF for downstream areas [50].

**Fig 8:**
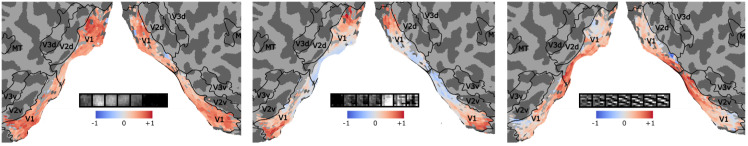
Projected U_*c*_ weight values for three different features in V1. Weight values were normalized between -1 and 1 by dividing them by the absolute maximum. The figure shows that the features are not evenly distributed across different cortical locations. The **U**_*c*_ matrix makes their analysis accessible.

Finally, we can investigate how stimulus features are encoded by investigating **U**_*c*_. In Fig 8 we show the feature weights for three different features in V1. We observe that different areas of early visual cortex show inhibition or excitation for the selected features. This provides insight into how stimulus features are represented across cortex.

**Fig 9:**
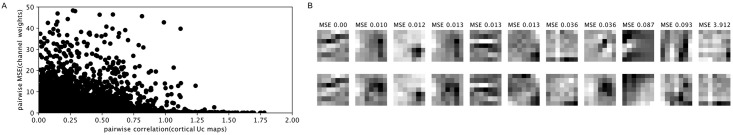
Relation between channel similarity and U_*c*_ map similarity. A. Relation between channel similarity and Uc map similarity in V1. Correlations are corrected with a fisher z-transform, and correlation signs are omitted. Highly similar Uc maps (high correlations) only occur for highly similar (small MSE) channels. However channels similar under MSE do not imply a highly similar Uc map. B. Examples of mean-squared error as a channel similarity measure.

It is of interest to examine whether these **U**_*c*_ weight distributions remain stable under different runs. We have run the same model five times, collecting the spatiotemporal channel weights and their associated **U**_*c*_ maps. Pairwise min-max-normalized mean-squared errors (MSE) were computed between these 5 × 64 channels to identify similar ones (low MSE implies similar channels, see Fig 9B for examples). The temporal dimension of the channels has been omitted by averaging over it as features appearing a few frames apart would have a large influence on the MSE, but little influence on Uc due to temporal pooling to TR. Likewise, we took pairwise Pearson correlations between the Uc weight maps (only significantly predictable voxels) for each channel, leading to 5 × 64 comparisons between approximately 1000 voxel-wise weights in V1. Signs of correlations were omitted as negative correlations between maps point at inverted weight maps which may occur as **U**_*c*_ is not constrained to be positive. Fig 9A shows the relation between both measures. While we do see that highly similar Uc maps only occur for highly similar channels, highly similar channels do not necessarily have highly correlated Uc maps. This analysis has been restricted to V1 as similar image-based comparison of higher order convolutional features is not possible.

### Processing of high-level semantic properties

So far, we have investigated characteristics of the NIF model that pertain to neural computations and representations and how these drive voxel responses. In this final analysis we investigate to what extent different neural populations are able to uncover high-level semantic content from the input stimulus. We focus on face detection since the processing of visual features pertaining to the discrimination of human faces is extremely well studied in the cognitive neuroscience literature [51]. In particular, FFA is known to play a central role in the visual processing of human faces [52]. Consequently, we expect that the representations learned by the FFA component of our model are related to human face processing.

**Fig 10:**
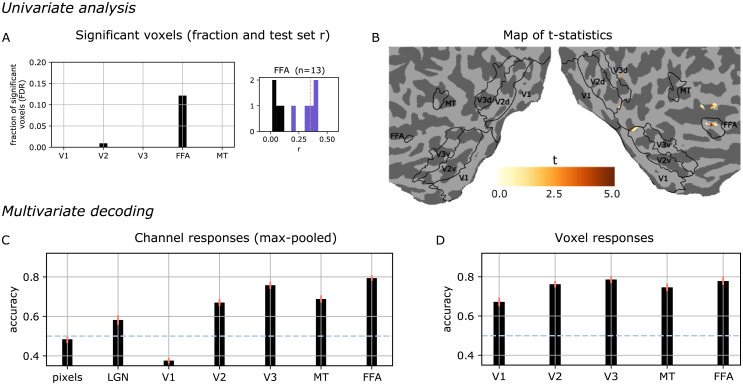
Results of an in-silico experiment. The trained network was presented with video segments from the test set showing either faces or no faces. A., B. Univariate analysis. A. Significant voxels in each ROI. Correlations between predicted and observed voxel responses on the test set. B. Cortical map of the t-statistic for univariate analysis. C., D. Multivariate logistic regression. C. Decoding from ROI-wise tensor activations (channel responses max-pooled across the whole feature map) or raw input values (*pixels*, *LGN*). D. Decoding from predicted voxel responses. Overall, we see that FFA is the most discriminative area for the face recognition experiment.

We test this hypothesis using an *in silico* experiment closely resembling standard fMRI experimental procedures in cognitive neuroscience. We passed 90 video segments of the regular input length of 3 TR, taken from the test set, through the trained NIF model. These videos were divided into two classes, one containing frontal views of human faces and the other not containing faces (45 videos per class). We analyzed the predicted BOLD responses of the models in the two experimental conditions using a mass univariate approach. For each voxel, we computed the t-statistic of the face minus no-face contrast and the associated p-values. We corrected for multiple comparisons using the false discovery rate (FDR) with alpha equal to 10^−4^. The left panel of Fig 10A shows the fraction of significant voxels in each brain region. The results show that FFA is the only region that is significantly activated by the contrast. The right panel shows that the voxels which are significantly activated also tend to be significantly predicted by the model. Fig 10B shows the significant (absolute) t-scores on the cortex.

We complemented these results with a multivariate decoding analysis [53]. We trained a logistic regression model on the predicted voxel responses of each ROI in order to predict if the input contained faces. We also performed this logistic regression analysis directly on the channel responses of the model (max-pooled across the spatio-temporal feature map). In the analysis we also included direct predictions from the pixel values of the input images. We estimated the mean accuracy and its standard error by repeating the training 50 times with random splits into 35 training and 10 test examples respectively. As shown in Fig 10, the highest classification performance is achieved for FFA, both at the channel level and at the voxel level. This confirms our expectation that the model FFA has learned higher-order semantic properties that match its functional role in the brain. Furthermore, we see that multivariate data from increasingly downstream regions are more suitable to dissociate faces from non-faces. This indicates the prospect of studying *in silico* what behavioural goals higher-order sensory areas are optimized for. This also hints at the possibility of using neural information processing systems estimated from brain data to support the solution of pattern recognition tasks.

### Data requirements

**Fig 11:**
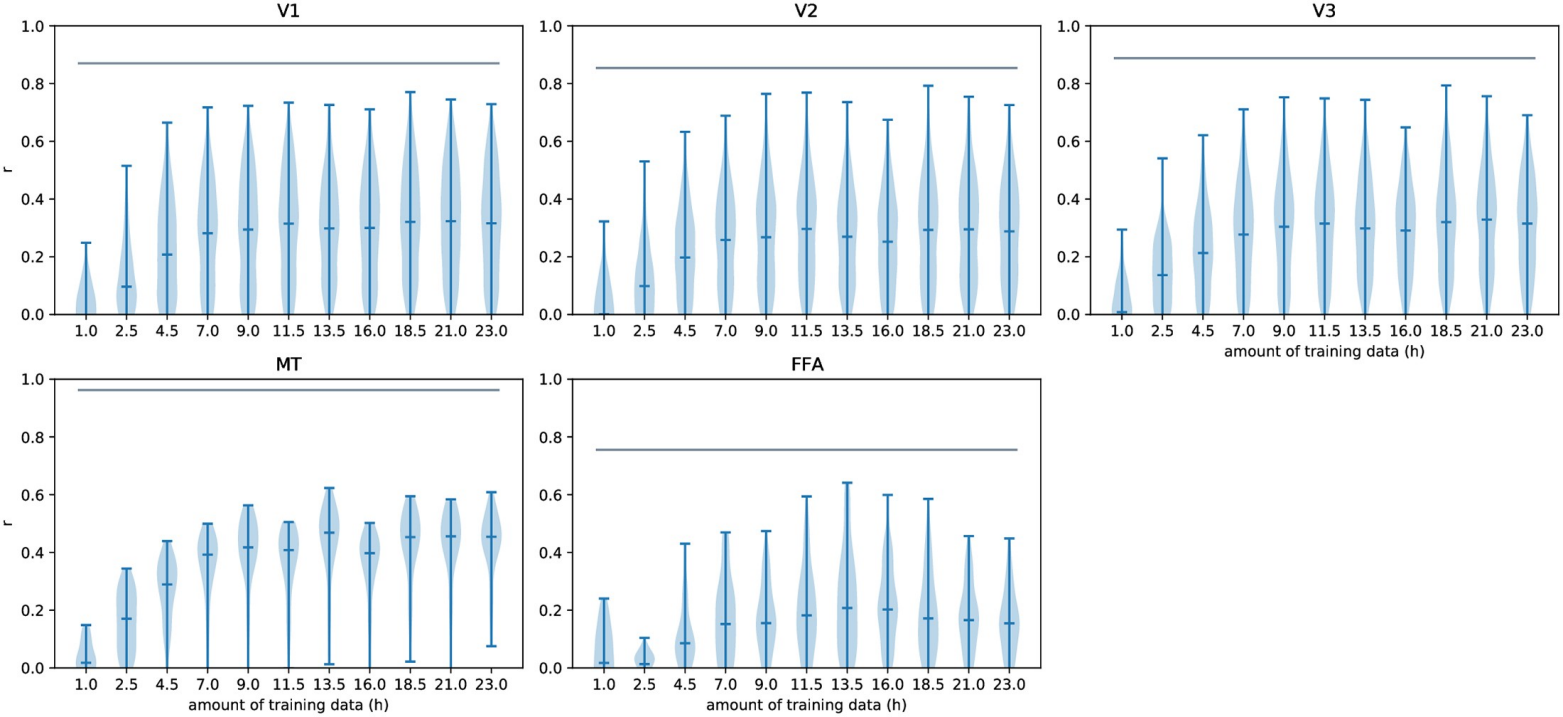
Test set performance over different amounts of training data. The example model was trained with increasing amounts of data, starting from the initial session. Voxel-wise correlations were determined on the test set for different areas, their distributions shown here. The performance of the example model saturates around the 12 hour mark. This result is likely specific for the stimulus modality, recording parameters, the model architecture and our particular participant.

The training of modern convolutional neural networks is known to require large amounts of data. The modeling framework described here likewise has data requirements that are not fulfilled by the large majority of current neuroscientific experiments. The required amount of data for a saturating model is unclear however. Fig 11 describes the data requirements for the specific experiment presented here. The example model we present saturates around the 12 hour mark. As several factors influence the required amount of training data this should neither be understood as a lower nor a higher bound on the amount of data required for applying this method. In general, we recommend to record single runs until test performance saturates.

The upper bars show the ROI-wise median of the voxel-wise noise ceiling of the correlation. It is an estimate of the upper limit on any model’s predictability attainable on the repeated test data set, given the noise in the data. An early description of the idea behind the noise ceiling can be found in [54]. We have used the Monte Carlo noise ceiling (MCnc) method mentioned in [55] and [56], and described in more detail in [57]. We have used Kendrick Kay’s public implementation. In the MCnc method, for every individual voxel, median correlations between simulated measurements and signals are estimated in a Monte Carlo simulation setting. Here a *measurement* is the sum between a signal and a noise component. *Signal* and *noise* are assumed to follow Gaussian distributions, for which mean and variance parameters are estimated from the z-scored data. The signal mean is the mean across the averaged test data time course. The noise mean is assumed to be 0. The noise variance is estimated across all test data repetitions. The signal variance is the rectified (non-negative) difference between the variance across the averaged test data time course and the noise variance. Using these parameters we have performed 500 signal simulations with 22 measurements (same signal, different noise) each. The figure shows the median of the voxel-wise noise ceilings within individual ROIs.

### Comparing to the task-driven approach

The currently most used technique for describing visual and auditory hierarchies is task-driven modeling with convolutional neural networks. A hypothesized convolutional neural network architecture is trained on a dataset with a specific objective function. Then experimental stimuli are passed through this pretrained architecture to obtain layer-wise activities in response to these stimuli, and the activity tensors are compared to brain activity under the same stimuli with encoding models or RSA. With these methods, layer distributions are identified across cortex. Many correspondences between modern convolutional neural networks and the visual system could be uncovered using the task-driven method.

Our aim with this paper is not to rival the currently best models in this area of visual modeling, but to propose a new approach to computational modeling of neural processing systems with a simplified visual system architecture as an example. Nevertheless we would like to attempt comparing quantitative performance between the task-driven and a data-driven approach using greedy readout models in the human visual information processing system. For video stimuli experiments it is common to use convolutional neural networks trained on video action classification [10]. We chose the R(2+1)D architecture [58], a well-performing network developed for action recognition on the Kinetics data set [59], based on ResNet [60]. It is a modern neural network architecture, including typical modern model choices like skip connections, batch normalization, ReLU units; and utilizing complex convolutional blocks with separated temporal and spatial convolutions. The network, originally trained on 15 Hz Kinetics data was fine-tuned on converted 22.86 Hz Kinetics data to align the learned temporal dynamics with our own data. The original model classified on cropped spatial windows inside the 112 × 112 × 16 data, which we omitted during fine-tuning to keep the input fixated around the fovea as in our NIF example model. The other training settings were kept identical to the description in the original paper and in the code, with the pretrained model published in pytorch torchvision [61].

**Fig 12:**
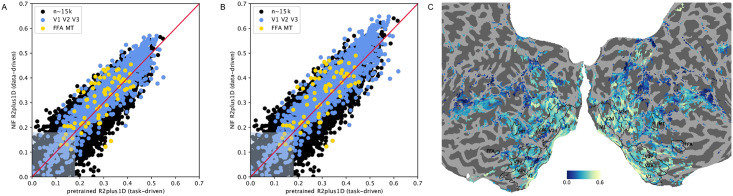
Comparison between task-driven and data-driven approach on our dataset. A. Correlations for early visual system and higher order areas. B. Correlations for early visual system and higher order areas (fisher-z corrected for linear comparability). Shaded areas cover non-significant voxels. C. Areas analyzed in this comparison, and their projected correlations.

Approximately 15.000 voxels with highest variance during the test set recordings were selected for this analysis, a number chosen in order to cover most of the visual system (see Fig 12C). We compared the task-driven case, using features pretrained on Kinetics; and the purely data-driven case, training all network parameters (convolutional features) on the objective function of predicting brain activity as in the NIF framework (thus denoted NIF in the figure). In both cases activity of all voxels was predicted based on the activity tensors conv1 to conv5 separately and in the same model. In the task-driven case, **U**_*s*_ and **U**_*c*_ readout parameters were learned for every voxel and layer, while the fixed pretrained features acted as a basis. In the data-driven case, both readout parameters and all convolutional block features were learned. RGB input was used, and the z-standardisation normalization used during pretraining was applied in the task-driven case as otherwise its performance would have been lower. The temporal dimension was omitted as R(2+1)D expects 16 frames. At 22.86 Hz this matched the number of frames shown in 1 TR of our data, so this merely restricted the model to predicting voxel-wise activity from video covering 1 TR instead of 3 TR, and not learning **U**_*t*_ parameters. To obtain voxel-wise correlations to estimate model performance, after model training for every voxel we chose the top-performing layer on the test set.

Results are shown in Fig 12. The task-driven and the data-driven approach are similar in performance, but the data-driven NIF-based approach outperforms the task-driven one using pretrained features especially in the early visual system and in higher order ROIs.

As the correlations achieved by the task-driven model are still relatively high and similar to the purely data-driven model our result only slightly contradicts the result of [13], where the predictive power of the pretrained features performed slightly better in V1. Potential explanations for these differences include that the R(2+1)D convolutional neural network does not match brain hierarchies well, however we do see a visible improvement in the data-driven case. Another explanation for these differences is that the cranial window in [13] has been on an area where pretrained DNN features indeed match V1 feature detectors well. Another different explanation is that the higher resolution of electrophysiological recordings leads to more accurate results than our functional MRI data.

This model comparison will not rule out the possibility that the pretrained features can be improved upon by using newer model developments from the machine learning community, or a more brain-like task. This numerical performance comparison should not distract the reader from recognizing the fundamental difference between the task-driven and our suggested data-driven modeling approach. By imposing an architecture of ROIs instead of taking the greedy approach, implemented as separate convolutional layers; we expect to learn the information processing between ROIs. A numerical performance comparison for this idea of training end-to-end models representing visual system architectures does not exist yet. Also, for sensory systems we believe it is worth exploring whether the data-driven approach leads to more accurate ROI representations, especially in higher order areas which divide into specialized areas solving different tasks important for human cognition—not all of which are known, and some of which may not be describable by neural network objective functions.

## Discussion

This paper proposes neural information flow for neural system identification. The approach relies on neural architectures described in terms of interacting brain regions, each performing nonlinear computations on their input. By coupling each brain region with associated measurements of neural activity, we can estimate neural information processing systems end-to-end. Using fMRI data collected during prolonged naturalistic stimulation we showed that we can successfully predict BOLD responses across different brain regions. Furthermore, meaningful spatial, temporal and feature receptive fields emerged after model estimation. The learned receptive fields are specific to each brain region but collectively explain all of the observed measurements. To the best of our knowledge, these results demonstrate for the first time that biologically interpretable information processing systems consisting of multiple interconnected brain regions can be directly estimated end-to-end from neural data.

As explained in the introduction, NIF generalizes current encoding models. For example, basic population receptive field models [62] and more advanced neural network models [5] are special cases of NIF that assume no interactions between brain regions and make specific choices for the nonlinear transformations that capture neuronal processing.

The researcher can specify alternative NIF models and then use explained variance as a model selection criterion. This is similar in spirit to dynamic causal modeling (DCM) [63]. However, NIF models can identify changes in neural computation that are not detectable in approaches that only focus on estimating effective connectivity. For example, they can be used to investigate in detail the changes in neural information processing under different conditions.

NIF can be naturally extended in several directions. The employed convolutional layer to model neural computation can be replaced by neural networks that have a more complex architecture. For example, recurrent neural networks can be trained in the same way as the feed-forward architecture presented here. Furthermore, lateral and feedback processing is easily included by adding additional links between brain regions and unrolling the backpropagation procedure over time. NIF models can also be extended to handle other data modalities. Alternative observation models can be formulated that allow inferring neural computations from other measures of neural activity (e.g., single- and multi-unit recordings, local field potentials, calcium imaging, EEG, MEG). Moreover, NIF models can be trained on multiple heterogeneous datasets at the same time, providing a solution for multimodal data fusion. The framework can also be applied to other sensory inputs. For example, auditory areas can be trained on auditory input (see e.g. [64]). If this is combined with visual input then we may be able to uncover new properties of multimodal integration [65].

Note that we are not restricted to using neural data as the sole source of training signal. We may instead (or additionally) condition these models on behavioral data, such as motor responses or eye movements [23]. The resulting models should then show the same behavioral responses as the system under study. We can also teach NIF models to perceive and act upon the task at hand directly using reinforcement neural network training [66]. In this way, NIF models provide a starting point for creating brain-inspired AI systems that more closely model how real brains solve cognitive tasks.

Finally, we can use NIF models as *in silico* models to examine changes in neural computation. For example, we can examine how neural representations change during learning or as a consequence of virtual lesions in the network [67]. This can provide insights into cognitive development and decline. We can also test what happens to neural computations when we directly drive individual brain regions with external input. This provides new ways for understanding how brain stimulation modulates neural information processing, guiding the development of future neurotechnology [68].

Summarizing, we view NIF as a way to construct biologically-inspired computational models that capture neural information processing in biological systems. As such, it provides a blend of computational and experimental neuroscience [69]. This gives us a principled approach to make sense of the high-resolution datasets produced by continuing advances in neurotechnology [70]. We expect that NIF models will deliver exciting new insights into the principles and mechanisms that determine neural information processing in biological systems.

### Code accessibility

A basic implementation of the NIF method on a smaller data set [71, 72] can be found at github.com/kateiyas/basicNIF.

## Supporting information

S1 Video Features (weights) learned inside the neural network layer for V1. (GIF)

S2 Video Animated preferred inputs for voxels in specific ROIs. (GIF)
