# Supplementary figures and images for: End-to-end neural system identification with neural information flow
Source: PLoS Comput Biol. 2021 Feb 4;17(2):e1008558. doi: 10.1371/journal.pcbi.1008558 (PMC7888598; doi:10.1371/journal.pcbi.1008558)

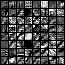

Supplement: S1 Video — (GIF) [file pcbi.1008558.s001.gif]

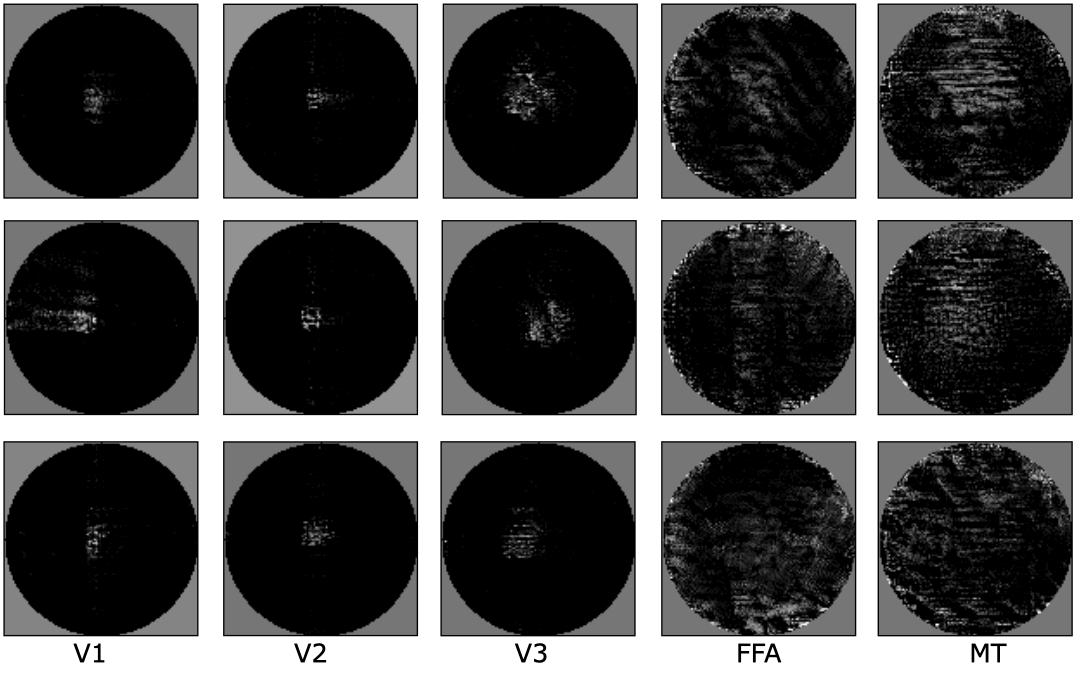

Supplement: S2 Video — (GIF) [file pcbi.1008558.s002.gif]
